# Immunogenicity and Lupus-Like Autoantibody Production Can Be Linked to Each Other along With Type I Interferon Production in Patients with Rheumatoid Arthritis Treated With Infliximab: A Retrospective Study of a Single Center Cohort

**DOI:** 10.1371/journal.pone.0162896

**Published:** 2016-09-19

**Authors:** Yuki Ishikawa, Takao Fujii, Seiko Kondo Ishikawa, Naoichiro Yukawa, Motomu Hashimoto, Moritoshi Furu, Hiromu Ito, Koichiro Ohmura, Tsuneyo Mimori

**Affiliations:** 1 Department of Rheumatology and Clinical Immunology, Graduate School of Medicine, Kyoto University, Kyoto, Japan; 2 Department of the Control for Rheumatic Diseases, Graduate School of Medicine, Kyoto University, Kyoto, Japan; 3 Department of Orthopedic Surgery, Graduate School of Medicine, Kyoto University, Kyoto, Japan; JAPAN

## Abstract

Besides anti-drug antibodies, anti-nuclear antibodies and anti-DNA antibodies are often induced in patients with rheumatoid arthritis treated with tumor necrosis factor inhibitors. We examined the association between immunogenicity, autoantibody production, and serum cytokine profiles in patients with rheumatoid arthritis treated with infliximab. Japanese patients with rheumatoid arthritis (n = 57) were retrospectively examined. Serum trough levels of infliximab, anti-drug antibody, anti-nuclear antibody, and anti-DNA (Farr), anti-single-stranded DNA and anti-double-stranded DNA antibodies were measured. Interleukin-6, interferon-γ, interferon-α, and B-cell activating factor levels were also measured in the same sera. Then, we validated the association between anti-drug antibody and these serum markers along with clinical response to infliximab. Anti-drug antibodies developed in twenty-one patients (36.8%), whose serum trough levels of infliximab were significantly lower than those in anti-drug antibody-negative patients (0.09 ± 0.03 vs. 2.48 ± 0.326 μg/mL, *p* < 0.0001). There were no significant differences in clinical backgrounds between the two groups. The anti-drug antibody-positive patients were more likely to develop anti-nuclear antibody titers of ≥ ×160 compared to the negative patients (14 to 57% vs. 17 to 33%). In addition, anti-DNA antibodies (Farr) (from 1.5 ± 0.4 to 35 ± 17 IU/mL, *p* = 0.0001), especially IgM-anti-double stranded DNA antibody (from 5.1 ± 0.7 to 41 ± 8.9 IU/mL, *p* < 0.0001), and IgG-anti-single stranded DNA antibody (from 13 ± 1.1 to 35 ± 13, *p* = 0.0145) were significantly increased in anti-drug antibody-positive but not in negative patients. Moreover, the anti-drug antibody-positive, but not the negative patients, showed significant increased levels of interferon-α (from 248.7 ± 102.3 to 466.8 ± 135.1 pg/mL, *p* = 0.0353) and B-cell activating factor (from 1073 ± 75.1 to 1387 ± 136.5 pg/mL, *p* = 0.0208) following infliximab treatment. The development of anti-drug antibody against infliximab and lupus-like autoantibody production in patients with rheumatoid arthritis treated with infliximab can be linked each other along with increased lupus-associated cytokine levels including type I interferons.

## Introduction

TNF inhibitors (TNFi) has been advantageous for many individuals with rheumatoid arthritis (RA). To date, five TNFis have been approved in Japan for the treatment of RA: infliximab (IFX), adalimumab, etanercept, golimumab, and certolizumab pegol. However, in some patients, an immune response is triggered by the TNFi, which results in the formation of anti-drug antibodies (ADAs). Immunogenicity is a harmful immunological reaction triggered by biological agents including TNFi. The prevalence of ADAs highly varies in studies of TNFi [1, 2], whereas the assay used for the detection of ADAs in a given study also influences the frequency of ADAs [3]. Development of ADAs is one of the causes of impaired clinical response to treatment with IFX or adalimumab in patients with RA [1, 4–10], and is also associated with increased frequency of clinical adverse events such as infusion reactions [11–13]. The development of ADAs have also been reported in other TNFi including etanercept [14–17], golimumab [18, 19] and certolizumab pegol [20–23], but the numbers of ADA-positive patients were too small to determine whether these ADAs were associated with an insufficient clinical outcome.

Autoimmune phenomena such as lupus-like syndrome or autoantibody production have also been observed in RA patients receiving TNFi, and some of these patients have shown insufficient clinical response [24]. However, the association between immunogenicity and autoimmunity has never been fully examined in RA patients; there has only been one study of such an association and that study showed a possible association between the two phenomena in psoriasis patients who received TNFi [25].

In the present study, we assessed IFX immunogenicity in Japanese RA patients treated with IFX and compared the profiles of autoantibody production before and after IFX treatment. We found that anti-nuclear antibodies (ANAs) and anti-DNA antibodies (Abs) were more frequently induced and their serum titers were higher in ADA-positive compared with ADA-negative patients. In addition, for the ADA-positive but not ADA-negative patients, the serum levels of interferon-α2 (IFN-α2) and B-cell activating factor (BAFF) were significantly increased after the initiation of IFX treatment, whereas the serum interleukin-6 (IL-6) and IFN-γ levels remained high levels. These data suggest that both ADA against IFX and lupus-like autoantibody production can be associated with each other, and type I IFN signals which might be driven by anti-TNF treatment can also impact on their developments.

## Materials and Methods

### Patients

Japanese RA patients treated with IFX at Kyoto University Hospital from 2004 to 2013 were initially picked up, and then patients whose serum samples were taken at least two time points, pre- and post-IFX treatment, were enrolled and retrospectively examined. All patients fulfilled the 1987 American College of Rheumatology classification criteria [26] and provided written informed consent. Patient information was extracted from electronic medical records. Stocked serum samples, which had been taken at multiple time points before and after the treatment, were used. The serum samples taken just before the first administration, 4 weeks after the initial treatment, and every 4 or 8 weeks thereafter were used. Serum samplings were missed at some points in some patients, and statistical analyses were performed on only available sera; the intention-to-treat approach was not used. For patients who discontinued IFX, but did not switch to the other biologic agents, sera obtained till originally scheduled next timing of IFX administration were used. For patients who switched to other biologics, sera obtained until next biologic disease modifying anti-rheumatic drugs (DMARDs) were administered were used. The Institutional Review Board of the Graduate School of Medicine, Kyoto University and Kyoto University Hospital approved the present study.

### Assessment of immunogenicity

Serum IFX trough levels and ADAs against IFX were measured using enzyme-linked immunosorbent assay (ELISA) and radioimmunoassay, respectively, at Sanquin Diagnostic Services (Plesmanlaan, CX, Amsterdam) [3].

### Autoantibody detection

ANAs and anti-DNA Abs (Farr) were measured using semi-quantitative indirect fluorescence assay and radioimmunoassay, respectively. IgG-anti-single-stranded-DNA (anti-ssDNA) and IgG-anti-double-stranded DNA (anti-dsDNA) Abs were measured by ELISA using MESACUP^™^ DNA-II test ss and ds (Medical & Biological Laboratories Co., Ltd., Nagoya, Japan), respectively. IgM-anti-dsDNA Ab was measured using ELISA (Alpha Diagnostic International, Texas, USA).

### Serum cytokine measurement

Serum cytokines were measured using the Milliplex MAP Multiplex Kit (Merck Millipore, Darmstadt, Germany) and obtained data were analyzed using Bio-Plex^™^ 200 (BIO RAD, Tokyo, Japan).

### Statistical analyses

All the serum parameters described above were measured using the same serum samples of given patients. Data are presented as mean ± SEM otherwise specified, and were compared using the Chi-square test, Fisher’s exact test, and the Mann-Whitney U test for two-group comparisons, and the Kruskal-Wallis test and Dunn’s multiple comparison test for multiple group comparisons. Spearmann’s correlation coefficient was calculated for changes of the obtained serum sample data and changes of DAS28-ESR. A log-rank test was used for assessment of the cumulative drug retention rate, and the proportion of ADA and ANA at different time points. *P* values < 0.05 were considered significant. All the statistical analyses were performed using GraphPad Prism 7 (GraphPad Software, Inc.).

## Results

### Study population

The clinical features of enrolled patients are summarized in Table 1. Fifty-five patients received IFX as a first biologic DMARD. We divided the patients into ADA-positive and ADA-negative groups; ADA positive group was consisted of patients who developed ADA during the observation, and ADA-negative group was consisted of patients who did not develop ADA throughout the observation. Only two patients were not biologic-naïve. One is included in the ADA-negative group with high disease activity, who previously received tocilizumab as a clinical trial and switched to etanercept, both of which were ineffective. The other is included in the ADA-positive group with high disease activity, who had previously received tocilizumab, and switched to IFX at the timing of the clinical trial end. ADA developed in 21 patients (36.8%) during observation. The median ADA titer was 96.0 (12–35,000) AU/mL. Mean duration of follow-up were 61.6 (12.8–117.9) and 65.3 (16.6–115.5) months in ADA-negative and ADA-positive group, respectively. There were no differences in patients’ backgrounds between the two groups. All the patients were administered with methotrexate (MTX); MTX doses did not significantly differ between the two groups. The frequency and doses of concomitant oral prednisolone were also not different between these groups. All patients had moderate to high disease activities; there was no difference in disease activity score 28-erythrocyte sedimentation rate (DAS28-ESR) between the two groups before the initiation of IFX treatment. Three patients had transiently withdrawn from IFX treatment for more than 3 months due to infection (pneumonia, sepsis) or suspected malignancy, and they later developed ADAs following re-administration of IFX (odds ratio 13.8, *p* = 0.046, Fisher’s exact test).

**Table 1:** Clinical features of the patients enrolled in this study.

	All patients	ADA (+)	ADA (-)	*P* value
Numbers of patients (%)	57	21 (38%)	36 (62%)	-
Age, years old	51.2±13.8	53.8±12.7	49.7±14.4	0.294^b^
Female (%)	45 (78.9)	16 (76.2)	29 (80.6)	0.927^c^
Disease duration	6.73±0.89	6.35±1.12	6.95±1.26	0.766 ^b^
Steinbrocker's Stage I+II (%)	58	66	53	0.819^d^
RF (U/mL)	262±158	89.7±15.4	372±258	0.559 ^b^
Sjӧgren’s syndrome	2	0	2	0.526 ^c^
MTX (%)	57 (100)	21 (100)	36 (100)	NS
MTX dosage (mg/week)	8.28±2.15	8.19±1.78	8.33±2.37	0.993 ^b^
sDMARDs except for MTX	9 (15.8)	3 (14.3)	6 (13.0)	1 ^c^
SASP (%)	5 (8.92)	1 (5.26)	4 (10.5)	0.642 ^c^
BUC (%)	1 (1.79)	0 (0)	1 (2.63)	1 ^c^
TAC (%)	3 (5.36)	2 (10.5)	1 (2.63)	0.548 ^c^
PSL (%)	29 (51.8)	11 (57.9)	18 (47.4)	1 ^c^
PSL dosage (mg/day)	3.03±4.18	3.64±3.88	2.67±4.35	0.443 ^b^
Initial DAS28-ESR	4.81±1.63	5.20±1.54	4.57±1.66	0.264 ^b^
As a 1st biologic (%)	55 (96.5)	20 (95.2)	35 (97.2)	1 ^c^
Discontinuation > 3 months^a^	3 (5.26)	3 (14.3)	0 (0)	0.046 ^c^
Mean duration of follow-up (months)	63.0	65.3	61.6	0.268 ^b^

^a^ Patients who discontinued IFX therapy for longer than 3 months.

^b^ Mann-Whitney U test,

^c^ Fisher’s exact test,

^d^ Chi-square test. Data are presented mean ± SD.

ADA, anti-drug antibody; RF, rheumatoid factor; MTX, methotrexate; sDMARDs, synthetic disease modifying anti-rheumatic drugs; SASP, salazosulfapyridine; BUC, bucillamine (penicillamine derivatives); TAC, tacrolimus; PSL, prednisolone. None of our patients were treated with hydroxychloroquine. DAS28-ESR, disease activity score 28-erythrocyte sedimentation rate

### ADA was associated with reduced clinical response

**Fig 1:**
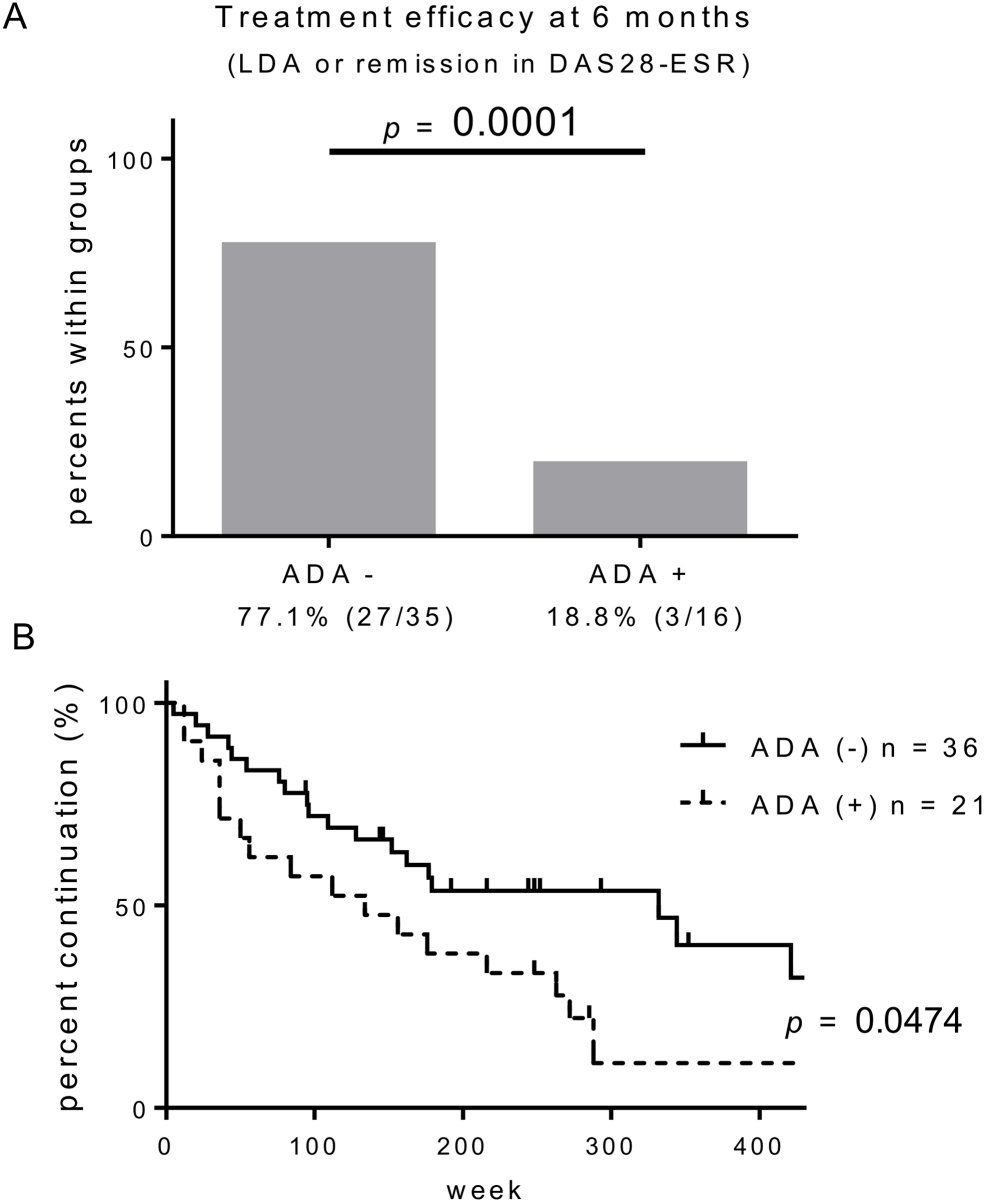
Anti-drug antibody (ADA) is associated with reduced clinical response. (A) Treatment efficacy defined as low disease activity (LDA) or remission at 6 months. Percentages and absolute numbers of each group of patients are indicated below the graphs. ADA positivity was based on the assessment of 6 months. Fisher’s exact test was used for comparison. (B) Cumulative drug retention rates. A log-rank test was used for comparison between the two groups.

ADAs were detected as early as 2.3 months after the initiation of IFX treatment. Of the patients who developed ADAs, 42.9% developed within the first six months and 80.9% developed within the first year of IFX treatment (Median 7.2 months). When we compared ADA-positive sera which showed seroconversion for the first time with ADA-negative sera which were taken at the time points (6.1 ± 1.3 months) when ADA-negative patients presented impaired clinical response, serum trough levels of IFX were significantly decreased in the ADA-positive sera compared with the ADA-negative sera (0.09 ± 0.03 vs. 2.48 ± 0.33 μg/mL; *p* < 0.0001). As had been reported previously, both the improvement of DAS28-ESR score at 6 months (-2.02± 0.26 ADA-negative vs. -1.27 ± 0.36 ADA-positive; *p* = 0.14) and the treatment efficacy, which was defined as low-disease activity (LDA) or remission, at 6 months were worse in the ADA-positive than in the ADA-negative group (Fig 1A). As a result, the cumulative drug retention rate of at 400 weeks was also lower in ADA-positive patients (Fig 1B). Primary or secondary failure was the most frequent cause of discontinuation of IFX treatment in both groups, whereas the rate of drug withdrawal by achieving remission was slightly higher in the ADA-negative group (S1 Fig). As was expected, an infusion reaction was observed in 3 patients, all of whom had ADA at the time of the event. Of the alternative treatments adopted after the development of ADAs, switching biologic agents gave the highest DAS28-ESR improvement (S2 Fig).

### ANAs developed more frequently in ADA-positive than in ADA-negative patients

**Fig 2:**
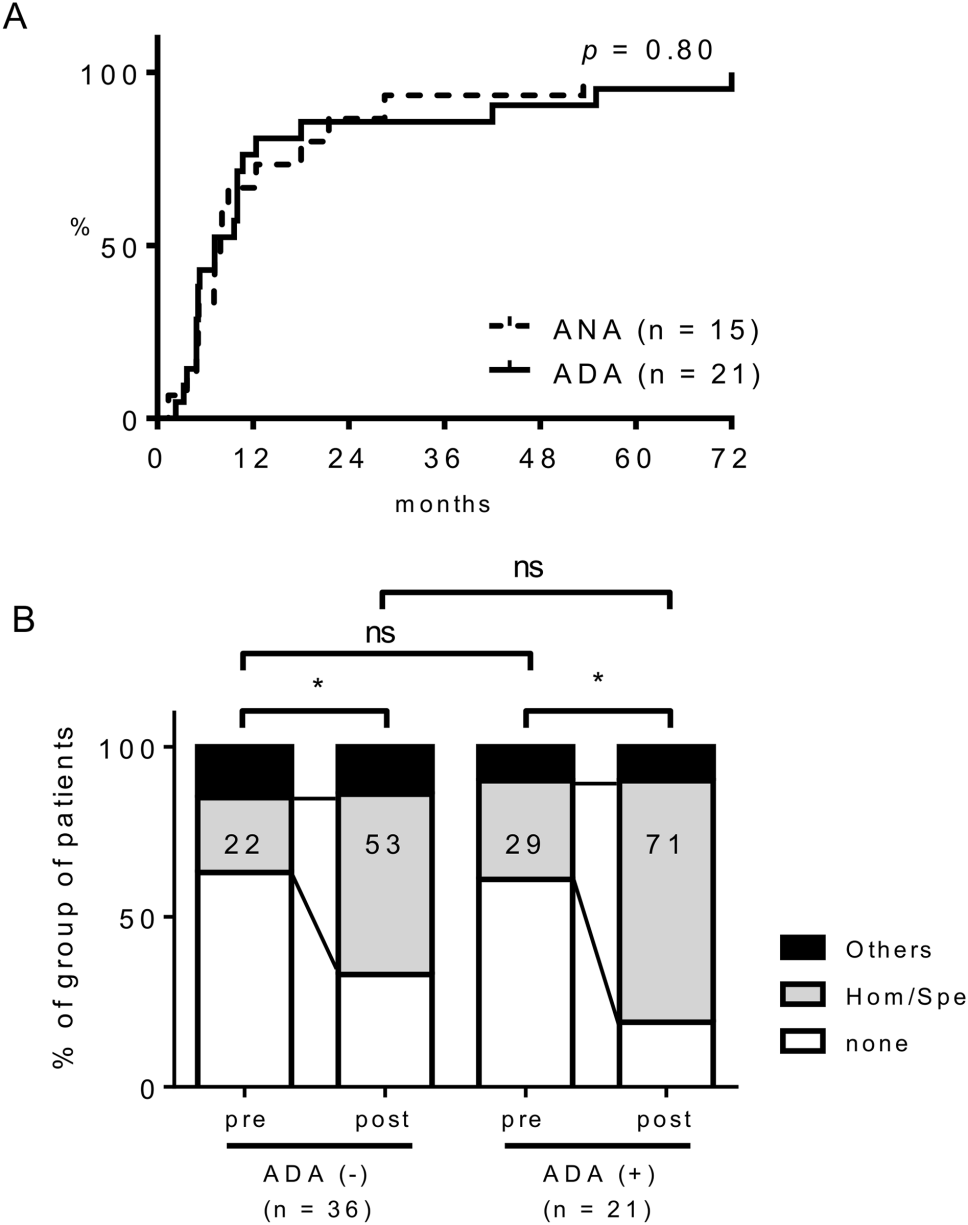
The association between ADA and ANA. (A) The proportion of ADA (n = 21) and ANA (**≥** ×160, n = 15) positive patients at different time points after IFX treatment in the ADA-positive group. A log-rank test was used for comparison between ADA and ANA curves. (B) Changes in the ANA staining pattern before (pre) and after (post) IFX. Numbers in bar graphs indicate percentages of patients with Hom/Spe pattern of FANA. None, ANA titer was ≤ ×40; Hom/Spe, homogenous/speckled. The chi-square test was used for comparison. * *p* = 0.037 (ADA-negative group) and *p* = 0.032 (ADA-positive group), ns: not significant.

To clarify the relationship between autoimmune phenomena and ADA formation, we first measured ANA titers in the sera before and after IFX treatment. The ADA-positive patients were more likely to develop ANA titers ≥ ×160 compared to the ADA-negative patients (14 to 57% vs. 17 to 33%). The average time for ANA titers to increase significantly, which was defined as at least doubled in consecutive measurements for patients with ≥ ×80 of base line ANA titers or at least quadrupled in consecutive measurements for patients with ≤ ×40 of base line ANA titers, from the initiation of IFX treatment was 15.4 ± 2.9 months. It is noteworthy that the median time to seroconversion was similar for ANA (7.9 months) and ADA (9.6 months) in the both ANA and ADA-positive patients (Fig 2A). The homogeneous/speckled pattern was the most frequently observed ANA staining pattern before IFX treatment. After the initiation of IFX treatment, most of the staining patterns turned to be homogenous/speckled pattern in both groups (Fig 2B). As homogenous/speckled pattern reflects the development of anti-DNA or anti-extractable nuclear antigen (ENA) Abs, we measured anti-ENA Abs including lupus-specific autoantibodies, such as anti-Sm and anti-U1-RNP Abs; however, none of the patients developed these lupus-specific anti-ENA Abs as well as lupus manifestations during the observation periods. Although some patients in both ADA-positive and negative group had anti-SS-A/La, or anti-SS-B/Ro Abs, there was no difference in the frequency between the groups (4.8% in ADA-positive group vs 8.3% in ADA-negative group; *p* = 1.00, Fisher’s exact test), and none of the patients developed these Abs after the initiation of IFX treatment.

### Anti-DNA Abs were more likely to develop in ADA-positive patients than in ADA-negative patients

**Fig 3:**
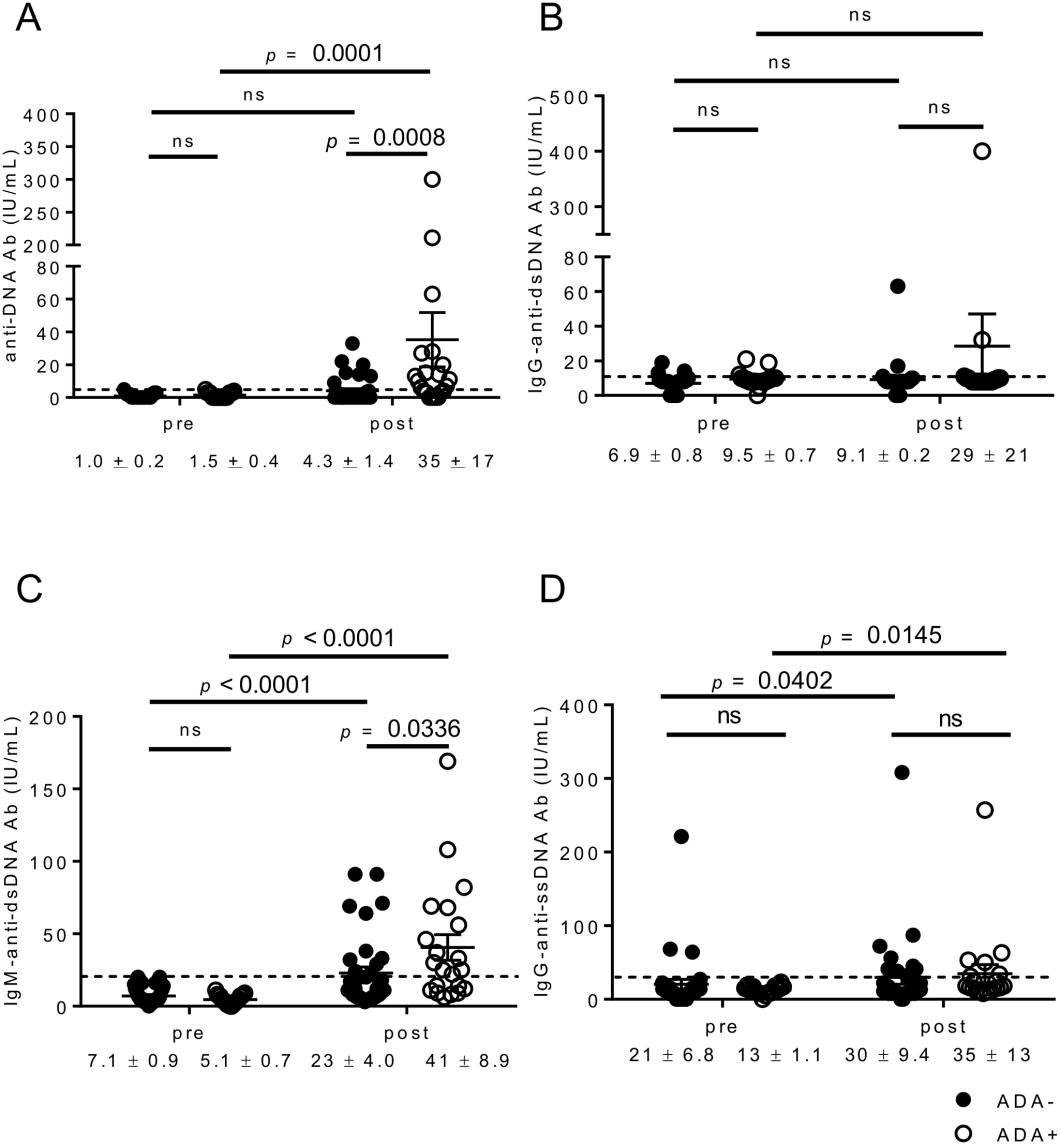
The association between ADA and anti-DNA Ab. The baseline (pre) and the peak (post) values of anti-DNA Ab (Farr) (A), IgG-anti-dsDNA Ab (B), IgM-anti-dsDNA Ab (C), and IgG-anti-ssDNA Ab (D). The upper limit normal values are indicated by dashed lines. The post values are the highest titers observed during the follow-up periods. Each dot represents a single serum sample, and the data are presented as mean ± SEM. A paired *t*-test for intra-group comparison or the Mann-Whitney test for inter-group comparison was used. ns: not significant.

We next measured anti-DNA Abs (Farr) in the pre- and post-IFX sera. The ADA-positive sera were more likely to show abnormal anti-DNA Ab (Farr) levels (upper limit of normal value 6 IU/mL) than the ADA-negative sera (12/21 (57%) vs. 8/36 (22%), *p* = 0.011, Fisher’s exact test) during the observation periods. Moreover, the ADA-positive but not ADA-negative patients developed significantly increased levels of anti-DNA Ab (Farr) during IFX treatment, and the highest post-treatment levels were significantly higher compared with the ADA-negative patients (Fig 3A). Interestingly, there was no difference in the levels of IgG-anti-dsDNA Ab between the two groups (Fig 3B). As the Farr assay detects both IgG and IgM Abs [27], and several groups have reported that TNFi induces IgM-anti-dsDNA Abs [28, 29], we measured IgM-anti-dsDNA Ab levels in the same sera (Fig 3C). Although both the ADA-positive and ADA-negative patients developed significantly increased levels of IgM-anti-dsDNA Ab after the initiation of IFX treatment, post-treatment levels were higher in the ADA-positive patients. In addition, IgG-anti-ssDNA Ab levels were also significantly increased after the initiation of IFX treatment in the ADA-positive group, although the difference of Ab titers between the groups was not significant (Fig 3D). Moreover, we found weak but positive correlations between ADA titers and titers of ANA (*r* = 0.282, *p* = 0.037), anti-DNA Ab (Farr) (*r* = 0.229, *p* = 0.0014), IgM-anti-dsDNA Ab (*r* = 0.277, *p* = 0.0002), and IgG-anti-ssDNA Ab (*r* = 0.155, *p* = 0.026). We also found that anti-DNA Ab (Farr) titers correlated better with IgM-anti-dsDNA Ab titers (*r* = 0.500 *p* = 0.0001) than IgG-anti-dsDNA Ab titers (*r* = 0.246, *p* = 0.0026), which further supports that the increased levels of serum anti-DNA Ab (Farr) reflected the increase of IgM-anti-dsDNA Ab in our cohort.

### Type I IFNs are also increased in the sera of ADA-positive patients

**Fig 4:**
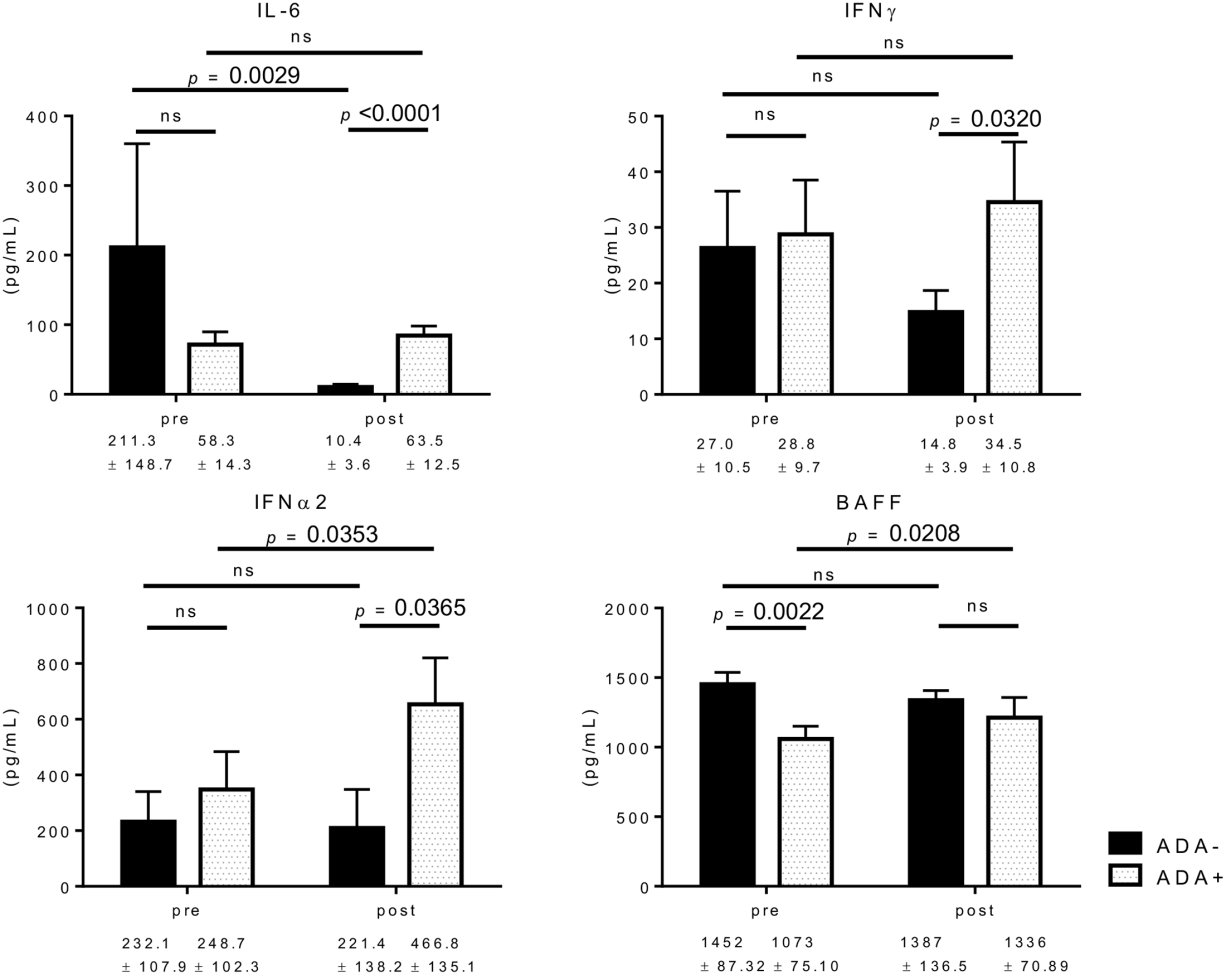
Serum cytokine levels before and 12 months after IFX treatment. The pre- and post-treatment levels of IL-6, IFN-γ, IFN-α2 and BAFF were compared between ADA-positive (+) and ADA-negative (-) groups. Data are presented as mean + SEM. A paired *t*-test for intra-group comparison and the Mann-Whitney test for inter-group comparison were used.

To investigate the underling mechanisms that can link immunogenicity and lupus-like autoantibody production, we measured the levels of various cytokines in the same sera in which the ADAs and autoantibodies were measured. We used sera taken 12 months after the initiation of treatment, as the most of ADA had developed within 12 months and ANA had also developed in the same timing (Fig 2A). There was no significant difference in the initial levels of cytokines we measured between the groups except for those of BAFF, which were significantly higher in the ADA-negative patients (Fig 4). Of the cytokines we measured, IL-6, IFN-γ, and IFN-α2 showed significantly higher levels in the ADA-positive than in the negative group after IFX treatment. Moreover, IFN-α2 levels were significantly increased after IFX treatment in the ADA-positive patients but not in the ADA-negative patients. One of the IFNα signatures, BAFF, also showed same trends as IFN-α2, although post-treatment levels were not different between the groups. While the ADA-positive patients kept high levels of IL-6 despite the IFX treatment, a significant decrease in IL-6 levels was observed in the ADA-negative patients following IFX treatment (Fig 4). In addition, changes of IL-6 level from pre-IFX to post-IFX positively correlated with DAS28-ESR changes, while IFN-γ and IFN-α2 changes did not. The changes of BAFF levels correlated with DAS28-ESR changes only weakly (S3 Fig). Intriguingly, post-treatment levels of both IFN-α2 and BAFF were higher in ANA-positive, than ANA-negative patients among ADA-positive group (S4 Fig), and IFN-α2 showed weak positive correlation with ADA (*r* = 0.314, *p* = 0.019) and ANA (*r* = 0.338, *p* = 0.012). Together these results indicated that type I IFNs can be linked to both immunogenicity and lupus-like autoantibody production in RA patients undergoing IFX treatment.

## Discussion

In the present study, we found an association between immunogenicity and lupus-like autoantibody production induced by IFX treatment of RA. To the best of our knowledge, this is the first report which describes the relationship between these immunological phenomena in RA patients treated by TNFi.

Patients who developed ADA showed markedly decreased trough levels of IFX, reduced clinical response, and poor cumulative drug retention rate compared to those who did not developed ADA; these data are comparable with previous reports [7]. Since all of the patients received MTX and the mean MTX dose did not differ between the two groups, we were unable to assess the influence of MTX on the immunogenicity of IFX in the present study. However, it is suggested that an MTX dose of up to 8 mg per week, which was the past-approved maximum dose in Japan, may have less impact on the immunogenicity of IFX compared to more than 8 mg per week of dosage. On the other hand, in the CONCERTO study, adalimumab concentrations increased with an ascending dose of MTX of up to 10 mg per week and treatment efficacy was equivalent between 10 and 20 mg of weekly MTX in combination with adalimumab [30]. Therefore, it may be speculated that at least 10 mg of weekly MTX is effective for preventing immunogenicity in IFX treatment. Although we did not measure baseline TNF-α levels, which would be useful to predict patients who need higher doses of TNFi to achieve enough trough levels of TNFi, it may worth studying whether baseline TNF-α levels affects the development of ADA against a given TNFi.

Previous data regarding the immunogenicity of IFX and adalimumab suggested that the majority of treated patients developed ADAs within the first 6 months of treatment [31]. On the other hand, most of our patients also developed ADAs within one year after the initiation of IFX treatment and half of them had developed ADAs within 6 months. Since this was a retrospective study and blood samples stored in the early phases of IFX treatment were limited, the frequency of ADA-positive patients might be higher in earlier phases after the initiation of IFX treatments than that suggested by the present data. Interestingly, the time to seroconversion was similar for ADA and ANA in the ADA-positive group (Fig 2A). This finding suggested that ADA might develop together with ANA in RA patients treated with IFX. This possibility is also supported by a recent study that showed an association between the seroconversion of ANA and secondary non-response to the TNFi [32].

The ADA-positive patients developed abnormal levels of serum ANA and anti-DNA Ab (Farr) more frequently than the ADA-negative patients after the initiation of IFX treatment. Since ADA-positive sera presented higher titers of anti-DNA Ab than ADA-negative sera (Fig 3A), the higher anti-DNA Ab titers are, the more likely ADA may develop although an absolute cut-off values are still unclear and correlation between ADA levels and anti-DNA Ab titers were weak. Both groups of patients developed IgG-anti-ssDNA Abs after the IFX treatment, which might partly explain the altered post-treatment expression of ANAs because the isotype of ANAs commonly measured is of IgG isotype. IgM-anti-dsDNA Ab, but not IgG-anti-dsDNA Ab, was also induced in patients who received IFX and was more significantly elevated in the ADA-positive patients. On the other hand, lupus-specific autoantibodies, such as anti-Sm or anti-U1-RNP Abs, were not developed in the patients, and none of the patients exhibited lupus-associated manifestations. These data indicated that the autoantibody induction observed in the present study was neither due to the concomitant existence of SLE in RA patients nor to a new onset of SLE, which included anti-TNF-induced lupus or drug-induced lupus [33]. It was previously demonstrated that most of anti-dsDNA Abs generated during the treatment of RA are of the IgM isotype [29, 34], and are not associated with other serological or clinically relevant signs of lupus [28]. On the other hand, the most prevalent isotype of ADA is of IgG isotype [3]. Therefore, an induced B-cell population that can further switch to IgG production may also have some roles in IgM-autoantibody production [29, 34], although further studies are needed to examine the exact roles of each Ig isotype and association among different isotype-producing B cells.

In the present study, we found that serum IFN-α2 was significantly increased in ADA-positive patients after IFX treatment. Type I IFNs, especially IFN-α, are known to be associated with various autoimmune diseases including SLE and are mainly produced by plasmatoid dendritic cells (pDCs) [35]. Autoantibodies associated with IFN-α production in SLE include anti-dsDNA Ab, as well as anti-RNA binding protein Abs [36]. Type I IFNs have also been suggested to be associated with Sjӧgren’s syndrome [37]; however, there was no difference in the frequency of Sjӧgren’s syndrome between the ADA-positive and ADA-negative groups in our cohort. It is also known that TNF-α inhibits the generation of pDCs and type I IFN secretion by pDCs, and in vitro culture of peripheral blood mononuclear cells with a TNFi increases IFN-α expression [38]. Moreover, a recent study of a murine model of TNFi-induced lupus, which is a TNF deficient mouse, showed constitutively increased numbers of circulating pDCs, developed high levels of autoantibodies, and type I IFNs and their related genes by chronic toll-like receptor-7-driven inflammation with pristane [39]. One of the IFN-α-regulated genes *BAFF* are known to be highly expressed in peripheral blood mononuclear cells of about 60% of adult SLE patients [40]. Although post-IFX levels were not different between ADA-negative and ADA-positive groups, the latter developed significantly higher levels of serum BAFF after IFX treatment. All these findings indicated that the levels of TNF-α and IFN-α (and its signatures) might influence the development of lupus-like autoantibody production following treatment with TNFi in humans. However, it is necessary to compare the changes of IFN-α2 and BAFF levels between IFX-continued and -discontinued groups in relation to ADA and ANA using enough number of patients in the future study to elucidate the relationships between serum cytokine imbalance and autoantibody production more clearly.

It is noteworthy that IL-6 induce the terminal differentiation of B cells into plasma cells and is important for plasma-cell survival [41], and type I IFNs induce IL-6 production from DCs [42]. Becker et al. reported that IFN-α2 levels were closely correlated with various cytokines including IL-6 in SLE patients with moderate activity [43]. The positive correlation between IL-6 and DAS28-ESR observed in our cohort indicated that increased IL-6 levels reflected increased RA activity, whereas higher levels of IL-6 in the ADA-positive group compared to the ADA-negative group might also reflect increased IFN-α2 activity. Moreover, It is known that T cells activated by type I IFNs are skewed towards Th1 immune responses with IFN-γ production [42]. Therefore, our results that the serum IFN-γ levels were higher in the ADA-positive than in the ADA-negative group after IFX treatment, and that there was no correlation between the levels of IFN-γ and DAS28-ESR, might indicate type I IFN-driven helper T-cell skewing in ADA-positive patients. Although we were not able to assess the cytokine levels at the time points earlier than 12 months due to the limited numbers of samples, cytokine levels at earlier time points will be necessary to define the underlining mechanism of both immunogenicity and lupus-like autoantibody production in the future study. In addition, as we were not able to evaluate any biomarkers described above in enough number of sera before seroconversion except for baseline in patients with ADA, it is also expected to evaluate sera just before seroconversion of ADA to delineate precise changes of these biomarkers.

Although RA patients who received less immunogenic TNFi, such as etanercept or golimumab, also developed lupus-like autoantibody production [32, 44], the frequency and relative odds ratio were higher in RA patients who received IFX or adalimumab compared to ones who received etanercept [45]. This fact also posts the possible association between immunogenic property of a given TNFi and development lupus-like autoantibody production.

We acknowledge that this was a retrospective study, the number of patients was limited, the doses of MTX and IFX used were lower than the doses used today, and lack of intention-to-treat approaches might cause bias to some degree. Furthermore, sera just before and after seroconversion of ADA were not able to be compared due to limited numbers of sera used in this study. Therefore, we must say that further prospective studies with enough number of patients are necessary to delineate the association between immunogenicity and lupus-like autoantibody production.

## Conclusions

We suggest that the development of ADA against IFX can be linked to lupus-like autoantibody production without clinical manifestations of lupus and subsequent insufficient clinical response. Moreover, type I IFNs might be involved in the production of both ANA and ADA in RA patients treated with IFX.

## Supporting Information

S1 Fig The causes associated with discontinuation of IFX. Failure included primary and secondary treatment failures. Other causes included infections, interstitial pneumonia, economical reasons. Chi-square test was used for two group comparison.
(TIF)

S2 Fig Comparison of DAS28-ESR improvements mediated by alternative treatments to IFX. Improvement in the DAS28-ESR score 6 months after the time of failure of IFX treatment in ADA-positive patients who were administered either 20 mg intravenous prednisolone (i.v. PSL) as premedication or increased doses of IFX (Dose-up; the mean dose of the last administration was 4.77±0.24 mg/kg), or whose treatment was changed to other biologic disease modifying anti-rheumatic drugs (Bio-switch) including etanercept (n = 4), adalimumab (n = 1), and tocilizumab (n = 3). Data are presented as mean ± SEM. Kruskal-Wallis and Dunn’s multiple comparison tests were used for comparisons. ns: not significant.
(TIFF)

S3 Fig Correlations between changes of cytokine levels (IL-6, IFN-γ, IFN-α2, and BAFF) and the changes of DAS28-ESR scores. The correlations between changes of cytokine levels shown in Fig 4 and the changes of disease activity score (DAS28)-erythrocyte sedimentation (ESR) were calculated for the same time point. Each dot represents data from a single patient. Spearmann r and approximate *p* values are indicated.
(PNG)

S4 Fig Post-IFX levels of IFN-α2 and BAFF. Post-IFX levels of IFN-α2 and BAFF levels were compared among ADA-negative with or without ANA and ADA-positive with or without ANA patients. Each dot represents data from a single patient. Data are presented as mean ± SEM. Mann-Whitney test was used for comparison.
(TIF)
