# Supplementary figures and images for: Immunogenicity and Lupus-Like Autoantibody Production Can Be Linked to Each Other along With Type I Interferon Production in Patients with Rheumatoid Arthritis Treated With Infliximab: A Retrospective Study of a Single Center Cohort
Source: PLoS One. 2016 Sep 19;11(9):e0162896. doi: 10.1371/journal.pone.0162896 (PMC5028026; doi:10.1371/journal.pone.0162896)

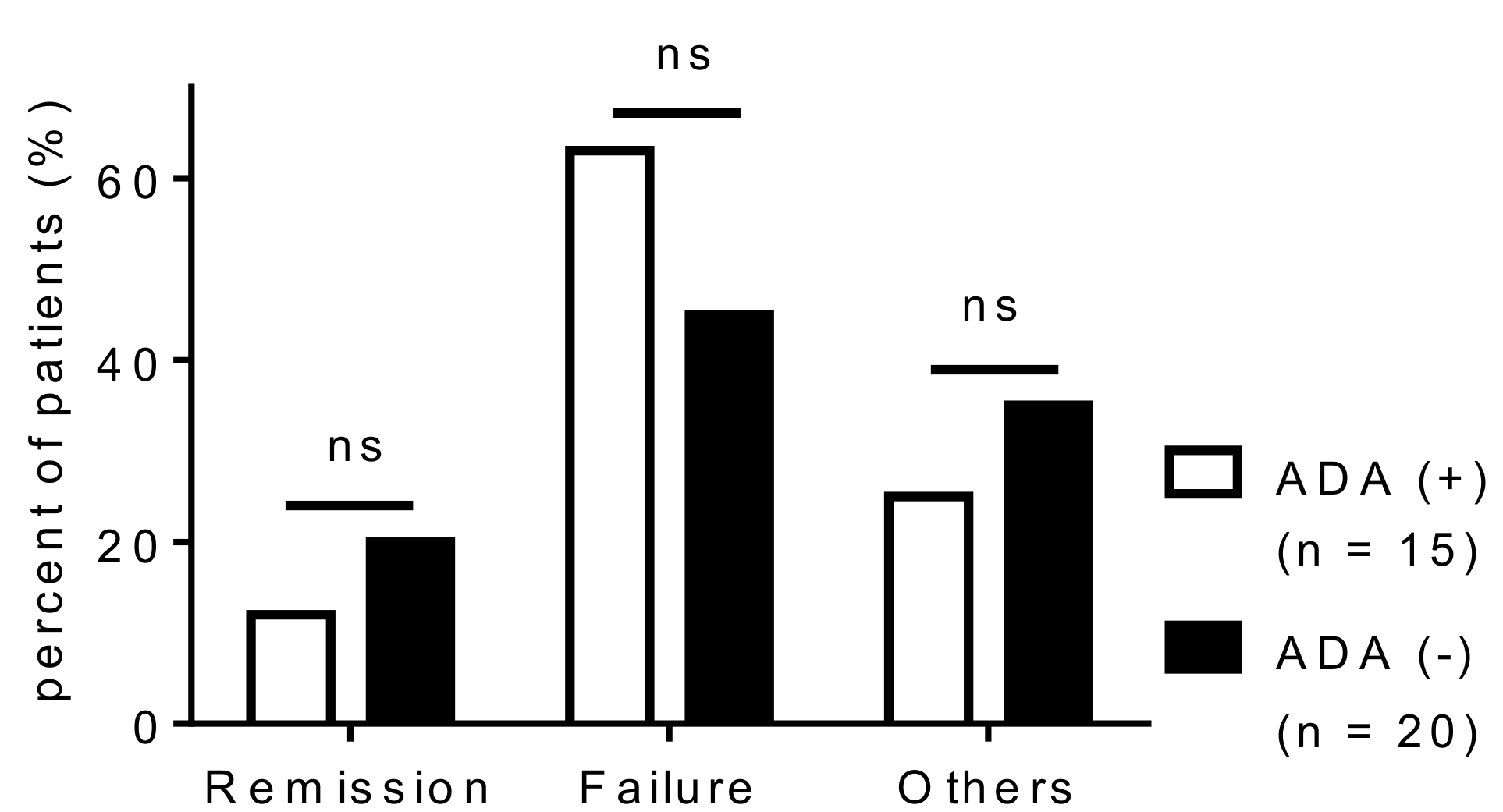

Supplement: S1 Fig — Failure included primary and secondary treatment failures. Other causes included infections, interstitial pneumonia, economical reasons. Chi-square test was used for two group comparison. (TIF) [file pone.0162896.s001.tif]

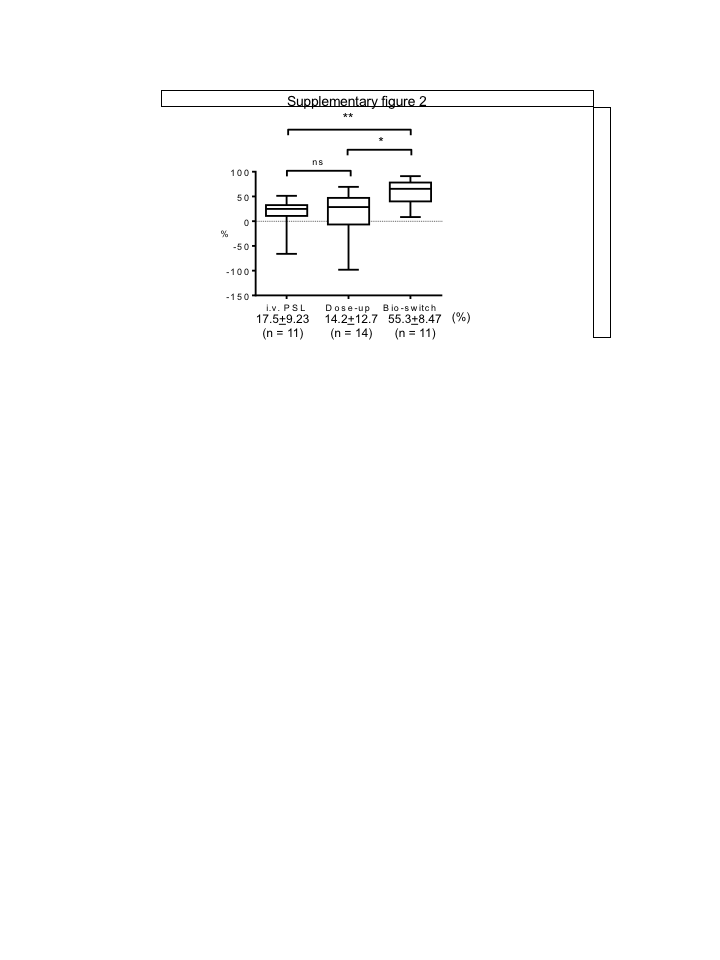

Supplement: S2 Fig — Improvement in the DAS28-ESR score 6 months after the time of failure of IFX treatment in ADA-positive patients who were administered either 20 mg intravenous prednisolone (i.v. PSL) as premedication or increased doses of IFX (Dose-up; the mean dose of the last administration was 4.77±0.24 mg/kg), or whose treatment was changed to other biologic disease modifying anti-rheumatic drugs (Bio-switch) including etanercept (n = 4), adalimumab (n = 1), and tocilizumab (n = 3). Data are presented as mean ± SEM. Kruskal-Wallis and Dunn’s multiple comparison tests were used for comparisons. ns: not significant. (TIFF) [file pone.0162896.s002.tiff]

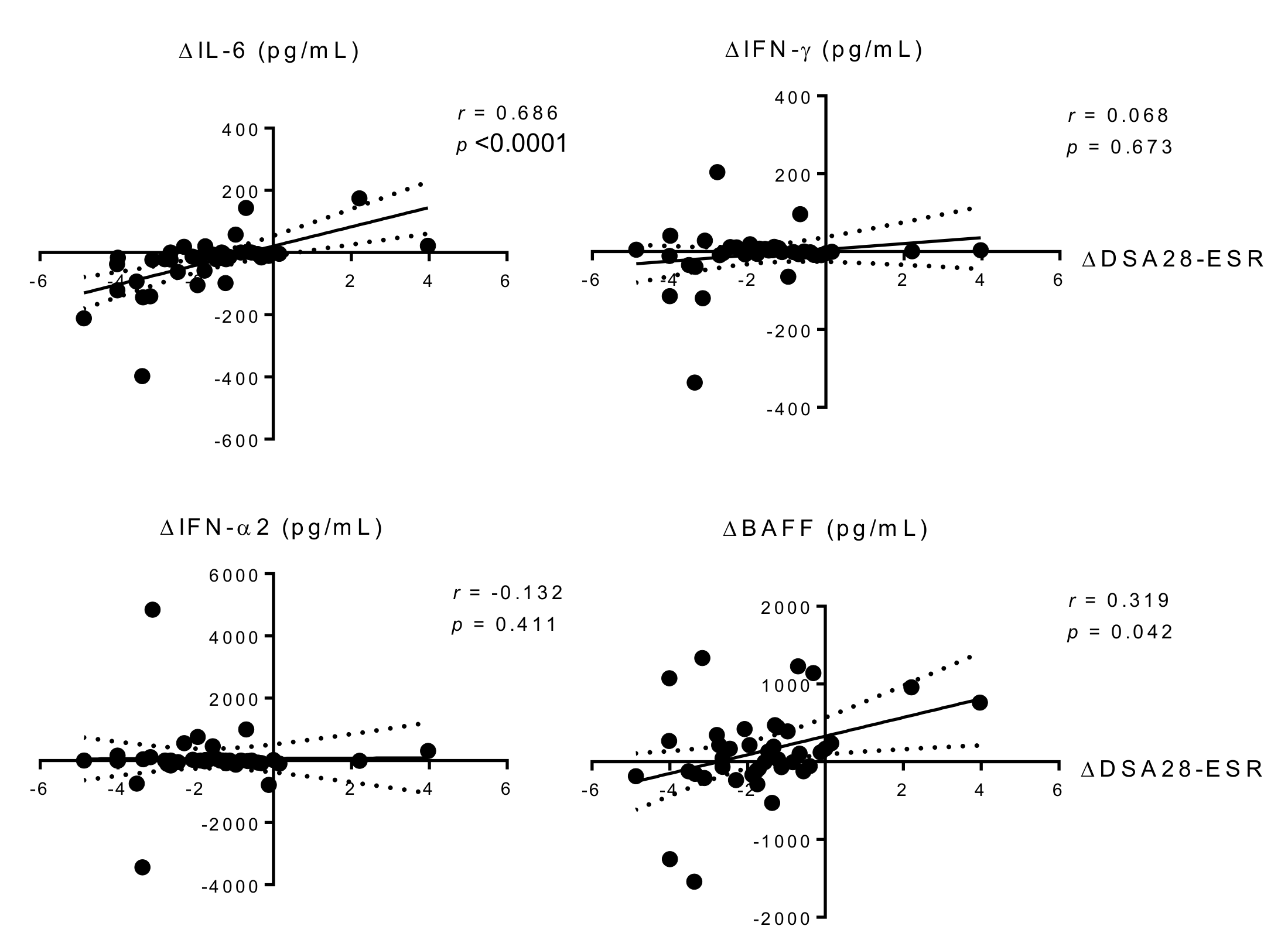

Supplement: S3 Fig — The correlations between changes of cytokine levels shown in Fig 4 and the changes of disease activity score (DAS28)-erythrocyte sedimentation (ESR) were calculated for the same time point. Each dot represents data from a single patient. Spearmann r and approximate p values are indicated. (PNG) [file pone.0162896.s003.png]

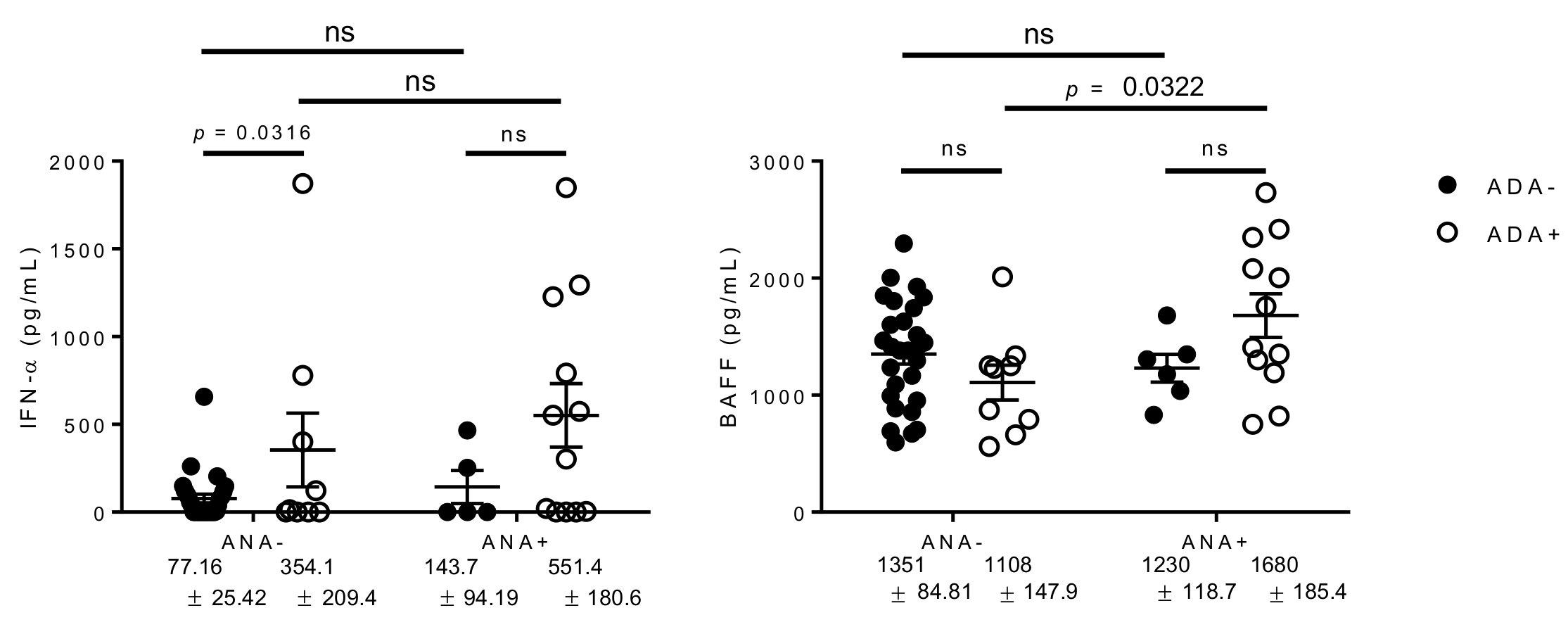

Supplement: S4 Fig — Post-IFX levels of IFN-α2 and BAFF levels were compared among ADA-negative with or without ANA and ADA-positive with or without ANA patients. Each dot represents data from a single patient. Data are presented as mean ± SEM. Mann-Whitney test was used for comparison. (TIF) [file pone.0162896.s004.tif]
